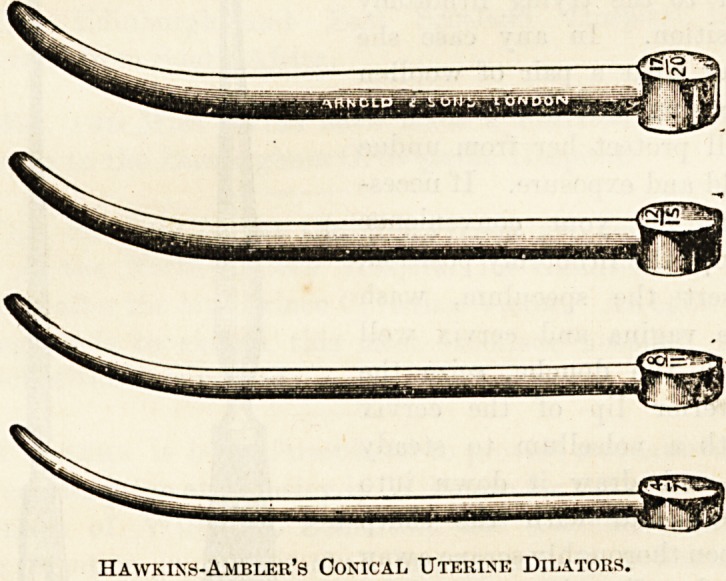# Treatment of Chronic Cervical Catarrh of the Uterus

**Published:** 1900-12-01

**Authors:** George A. Hawkins-Ambler

**Affiliations:** Surgeon to the Samaritan Hospital for Women, Assistant Surgeon, Stanley Hospital, Liverpool


### Hospital Clinics and Medical Progress.

### TREATMENT OF CHRONIC CERVICAL CATARRH OF THE UTERUS.

By George A. Hawkins-Ambler, F.R.C.S.E., Surgeon to the Samaritan Hospital for Women, Assistant Surgeon, Stanley Hospital, Liverpool.

In discussing the treatment of endocervical catarrh in a recent article, we saw that it was necessarily prolonged, but not of necessity radical. Cases occur, however, that call for energetic and radical treatment: patients in whom the cervical mucosa has become so diseased, its glands choked with their secretions, degenerated, obliterated, or represented by depressions, that replace ruptured follicles, while at times the papillæ of the mucosa are enlarged and hæmorrhagic. For these advanced cases we may try the prolonged application of pure phenol or iodised phenol by means of a small piece of lint dipped in it and passed into the cervix, while a good-sized tampon is placed below it to protect the vagina. If this be left in for a day or two the effect is often very satisfactory. On removing the dressing a douche may be given. The occasional application of the acid nitrate of mercury is effective in removing the affected tissues. Care must be taken to have no excess of the application on the dressed probe or skewer, and to carefully place a tampon beneath the cervix to prevent injury to the vagina. Similarly, a caustic stick, made by fusing equal parts of sulphate of alum and sulphate of zinc together, may be placed in the cervix and retained in position by a protecting pad of wool.

Another method of destroying the diseased mucosa is by curettage. We see a good many patients who have been "curetted," but the only indication of the performance of the operation is the subsequent cellulitis. Done without an anæsthetic, with no obvious antiseptic precautions, and no indication of anything having been effected save the introduction of sepsis, it is difficult to understand what some practitioners look upon as efficient curettage, and more easy to see why the operation should not be popular or successful with them.

To be useful, curettage must be thorough, systematic, and aseptic. In gynæcology, as in general surgery, one must be careful about the small operations and the great ones will take care of themselves. It is in the former that carelessness and indifference are shown to routine antiseptic precautions, which, if necessary anywhere, are essential in the vagina.

Curettage of the cervical endometrium is not a difficult or risky operation, provided one adopts intelligent precautions. I have done it in my consulting room, but it is better to do it at the patient's home and to keep her in bed a day or two according to the extent of your interference. The following preparation, too, may seem elaborate, but is by no means excessive. For a day or two before curetting the cervix, let your patient have antiseptic douches of, say, a quart of a 1 in 1,000 or 1 in 2,000 solution of a biniodide of mercury, or perchloride of mercury, or some other antiseptic twice daily. The douche must be given carefully, with the patient recumbent, and the vagina held open by the nurse's finger while a strong stream of lotion is played into it. An aperient may be taken the day before. An anæsthetic is scarcely required; but if the patient be sensitive, a small pledget of cotton dipped in a 15 to 20 per cent. solution of cocaine may be placed against the part to be curetted for a few minutes before attacking it. You will require a Sims' speculum; and I may say here that for operative purposes I use a speculum double the ordinary size and weight. This gives one more room and, when short-handed, usually suffices to keep the vagina open by its own weight. You will need also one or two volsellæ, preferably straight and locking with a catch; a narrow Volkmann's spoon or Récamier's uterine scoop;[1] two or three game skewers, Playfair's probes dressed with cotton, or dressing forceps armed with cotton; an antiseptic douche as above named; and a Clover's crutch.

Simon's Uterine Scoop. Thomas's Curette

For slight cases it will be sufficient to place the patient in the Sims' position, since it is not necessary to subject her to the trying lithotomy position. In any case she may wear a pair of woollen stockings and drawers, which will protect her from undue cold and exposure. If necessary for your convenience adopt the lithotomy position, insert the speculum, wash the vagina and cervix well with the douche, seize the anterior lip of the cervix with a volsellum to steady it, and draw it down into view, and with the sharp spoon thoroughly scrape away all the affected mucosa of the cervix. Wipe or douche away the débris; apply pure carbolic acid to the scraped tissues; dry again, and pack into the cervix lightly some double cyanide or iodoform gauze and leave a pad of it under the cervix to catch any excess of the caustic used. The operation is now complete. You will of course have boiled your instruments for half an hour before use in a weak soda solution, and the dressing must also be sterilised.

It will be well for the patient to spend a day or two in bed. Remove the gauze in 36 to 48 hours, administer an antiseptic douche, which repeat twice daily for a week, after which once a day will be sufficient. Give the woman an aperient, and resume the constitutional treatment you had adopted. The local applications may be confined to the antiseptic douches, varied with ichthyol tampons if there be much congestion, or douches of zinc or alum, and applications of more or less concentrated preparations of phenol if the cervix does not steadily resume its normal condition.

Cases occur where endocervicitis is complicated by extension of the mischief into the mucous lining of the body of the uterus, in which event there will be some roughness of the endometrium, with probably enlargement of the cavity, roughness of its walls, and profuse

B

menstrual flow. Or a further extension may be indicated by symptoms of salpingitis, &c. Here it will not be sufficient to curette the cervix; a general curettage of the uterus is called for, with still more careful preparation. In addition to the preliminary douching, the bowels should be emptied by an enema before operation. An anæsthetic will be required, the genitals should be shaved, scrubbed with soap and water and an antiseptic, and the lithotomy position adopted. The patient may be drawn to the edge of the bed, a mackintosh sheet placed beneath her and draped into a basin lying on the floor; or she may be placed on a table for the operation. I use the following instruments, &c.:—Clover's crutch; Sims' speculum; two volsellæ; my own uterine dilators, sizes 1 to 8 or 9; Simon's sharp spoon; Thomas's curette; 12 game skewers dressed with cotton; dabs of cotton; half a yard of double cyanide gauze in two strips; pure carbolic acid or iodised phenol. For a lubricant carbolised glycerine, 1 in 10, or Lund's oil does very well. As to the dilators, mine

Hawkins-Ambler's Conical Uterine Dilators.

are a modification of Hegar's, differing in being conical. They have the advantage over Hegar's that where one size has passed the next will always pass, while it occasionally happens that Hegar's, of which a much larger number are required, sometimes hitch. Increasing in complete sizes, there may be a difficulty in introducing following sizes. My dilators are on Hegar's scale, only my No. 1 commences at No. 3 Hegar—at the point—increasing three sizes in the first inch and a half (as they all do), and reaching a fourth size in the next inch. Being metal, they are easily cleaned and boiled.

[1] Arnold's Catalogue, Fig. 1,926.

(*To be continued.*)

## Figures and Tables

**Figure f1:**
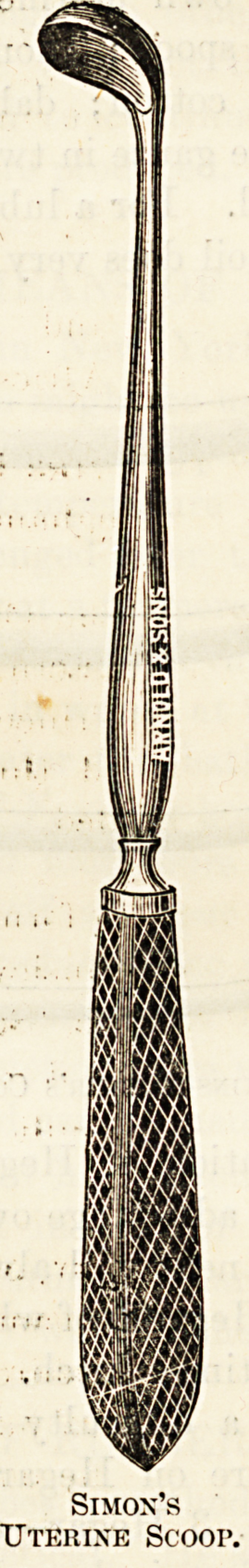


**Figure f2:**
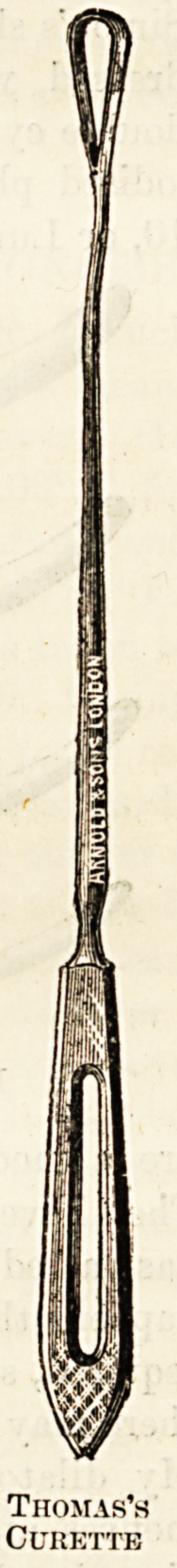


**Figure f3:**